# High-Throughput cell-based immunofluorescence assays against influenza

**DOI:** 10.1016/j.slasd.2023.10.008

**Published:** 2023-11-02

**Authors:** Yohanka Martinez-Gzegozewska, Lynn Rasmussen, Sara McKellip, Anna Manuvakhova, N. Miranda Nebane, Andrew J. Reece, Pedro Ruiz, Melinda Sosa, Robert Bostwick, Paige Vinson

**Affiliations:** Scientific Platforms Division, Southern Research, High-Throughput Screening Center, Birmingham, Alabama, United States

## Abstract

A rapid drug discovery response to influenza outbreaks with the potential to reach pandemic status could help minimize the virus’s impact by reducing the time to identify anti-influenza drugs. Although several anti-influenza strategies have been considered in the search for new drugs, only a few therapeutic agents are approved for clinical use. The cytopathic effect induced by the influenza virus in Madin Darby canine kidney (MDCK) cells has been widely used for high-throughput anti-influenza drug screening, but the fact that the MDCK cells are not human cells constitutes a disadvantage when searching for new therapeutic agents for human use. We have developed a highly sensitive cell-based imaging assay for the identification of inhibitors of influenza A and B virus that is high-throughput compatible using the A549 human cell line. The assay has also been optimized for the assessment of the neutralizing effect of anti-influenza antibodies in the absence of trypsin, which allows testing of purified antibodies and serum samples. This assay platform can be applied to full high-throughput screening campaigns or later stages requiring quantitative potency determinations for structure-activity relationships.

## 1. Introduction

Influenza is a small RNA virus and, as a consequence, there are only a few viral proteins that can be considered as targets for virus inhibition by small molecules. Among these targets are the neuraminidase, the M2 protein and the RNA-dependent RNA polymerase activity of PB1 and the endonuclease activity of PA [1].

Although several anti-influenza strategies have been considered in the search for new anti-influenza drugs [2], only a few therapeutic agents are approved/recommended for clinical use, pointing to the need for new assays that are compatible with a high-throughput screening (HTS) platform, allowing for a rapid drug discovery response to influenza outbreaks.

Table 1 summarizes cell-based HTS approaches developed for influenza antiviral discovery, with some of the corresponding challenges that create barriers to the process of identifying new anti-influenza drugs.

We have developed a highly sensitive imaging cell-based assay for the identification of inhibitors of influenza A and B virus using the A549 human cell line. It is high-throughput compatible and involves multiple virus infection and replication cycles allowing the identification of anti-influenza compounds that inhibit any stage of the virus life cycle by directly measuring the virus, thus combining the advantages of influenza CPE and reporter assays in a human cellular environment. The staining procedure has been reduced to the addition of a directly conjugated antibody after removal of the assay media. To overcome the challenge of the trypsin requirement to cleave the hemagglutinin (HA) of the new virus particles, we adapt the cells to grow in reduced serum media with 2 % fetal bovine serum (FBS) which is substituted in the assay media by a low percent of bovine serum albumin (BSA). A cytotoxicity counter screen is required to avoid the selection of cytotoxic compounds that, same as an antiviral compound, will produce a low signal due to the decrease in the number of cells.

The antiviral assay has also been optimized for the assessment of the inhibitory capability of anti-influenza antibodies in serum samples. The conditions have been adjusted for the virus to follow only one round of replication by infecting the cells in the absence of trypsin at a multiplicity of infection that is at least 10 times higher than needed for the multicycle assay.

This assay platform can be applied to full high-throughput screening campaigns or later stages requiring quantitative potency determinations for structure-activity relationships. Its application extends to any influenza virus, or even non-influenza viruses, when instrumentation and labeled antibody are available, enabling a capability for assessment of the antiviral activity of experimental compounds across a panel of different influenza and non-influenza viruses.

### Materials

#### a. Reagents

- OptiMEM I Reduce-Serum Medium no phenol red (Cat #11058021; Fisher Scientific)
- Fetal Bovine Serum Pure Premium Grade (Cat #PS-FB4; Peak Serum) heat inactivated at 56°C for 1 hour.
- Penicillin-Streptomycin (Cat # 15140122; Fisher Scientific)
- PBS (Cat # 10010-031; Fisher Scientific)
- TrypLE Express (Cat # 12604021; Fisher Scientific)
- Dimethyl sulfoxide (Cat #D8418; Sigma-Aldrich)
- Trypsin TPCK (L-1-tosylamido-2-phenylethyl chloromethyl ketone) treated (Cat #22725; Affimethix/USB products)

Trypsin is prepared 1 mg/mL in PBS plus Ca+/Mg+, 0.2 μm filtered for sterilization, aliquoted for single use and kept at −70 °C.

- Bovine serum albumin (Cat #A2153; MilliporeSigma,)

BSA is prepared 10 % in OptiMEM/1 % PS and 0.2 μm filtered for sterilization.

- Oseltamivir carboxylate (Cat #HY-13318), ribavirin (Cat #HY-B0434), Pimodivir/VX-787 (Cat #HY-12353A) were all purchased from MedChemExpress
- Benzethonium chloride or hyamine (Cat #53751; MilliporeSigma)

Hyamine is prepared 10 mM in DNase/RNase free distilled water and 0.2 μm filtered for sterilization.

- CellTiter-Glo (Cat # G7573; Promega)
- Influenza A antibody directly conjugated to a fluorophore.

The data shown in this manuscript has been obtained using Influenza A m2 (14C2)-Alexa Fluor 647 (Cat # sc-32238 AF647; Santa Cruz Biotechnology)

- Influenza B antibody directly conjugated to a fluorophore.

The data shown in this manuscript has been obtained using Influenza B antibody (1131 (2B2))-Alexa Fluor 647 (Cat #NB100-65034AF647; Novus Biologicals)

#### b. Cell lines

- Human Epithelial Lung Carcinoma (A549) cells (Cat #CCL-185; American Type Culture Collection) are used for high content antiviral assay.
- Mardin Darby Canine Kidney (MDCK) cells (Cat #84121903; Sigma-Aldrich) are used for growing of virus and titration experiments.

#### c. Influenza virus strains

A/WSN/33, H1N1 and A/Udorn/72, H3N2 were provided by Dr Robert Krug’s laboratory at the University of Texas in Austin. All other strains were obtained from ATCC and BEI resources and all are part of Southern Research inventory.

#### d. Microtiter plates

- Echo Qualified 384 well polypropylene plates (Cat # P-05525; Beckman Coulter)
- Axygen 384 well clear V-bottom 120 μL polypropylene deep well not treated plate (Cat # P-384-120SQ-C; Corning Life Sciences)
- Black clear bottom tissue culture treated collagen coated 384 well plates (Cat # 312003; J. G. Finneran)
- Black clear bottom tissue culture treated 384 well plates (Cat # 3764; Corning Life Sciences)

#### e. Equipment

##### Bulk liquid dispensers

- Wellmate (Matrix Wellmate, Thermo Scientific)
- Combi (Multidrop Combi, Thermo Scientific)

##### Liquid handlers

- Echo 555 acoustic liquid handler; Beckman Coulter
- Biomek FX Single Arm System with Multichannel Pipettor; Beckman Coulter

##### Robot systems

- Thermo Fisher Scientific Catalyst Express

##### Readers

- Mirrorball SPTLabtech
- PHERAstar FSX, BMG LABTECH GmbH

##### Other

- Spectrolinker XL-1500 (Spectro-UV)

#### f. Software

- IDBS ActivityBase and ActivityBase XE (both version 9.7) run on Windows OS
- ThermoScientific Momentum scheduling software

## 2. Procedure

Timing reflects the total length of each major step. Timing for specific stages of the procedure are shown in Table 2. Critical assay considerations/steps would apply to the procedure as a whole and hence will be described at the end of the procedure.

### Cell culture

#### Timing: ~5 weeks until cells are ready for the assay

As the addition of trypsin to the assay media is necessary for the proteolytic activation of the influenza HA to occur, the FBS needs to be removed from the assay media. Cells are then adapted to grow and culture in OptiMEM I Reduced-Serum Medium with a minimum percentage of FBS. The adaptation and culture methods apply to both cell lines used in this protocol, MDCK and A549.

##### 1. Adaptation of cells to grow in OptiMEM media

- Thaw a vial of cells and seed them in a T75 culture flask containing 15 mL of the media recommended by the provider. Either wash the cells with media before seeding or replace the media with fresh media the next day.
- Passage the cells 2 times in the media recommended by the provider and then start adapting them to grow in OptiMEM by gradually increasing the ratio of OptiMEM in the culture media, keeping the percentage of FBS at 10.
- Passage the cells 2 times in OptiMEM 10 % FBS and then reduce the percentage of FBS to 5.
- Passage the cells 2 times in OptiMEM 5 % FBS and proceed to harvest the cells for cryopreservation.
- Resuspend the pellet of cells in a volume of FBS/5 % DMSO to have 1×10^6^ cells/mL.
- Make 1 mL aliquots and rate freeze the vials at −80°C before storing them in a liquid nitrogen tank.

##### 2. Culture

- Thaw a vial of cells and add them to a T75 culture flask containing 15 mL of OptiMEM/5 % FBS. Either wash the cells with media before seeding or replace the media with fresh media the next day.
- Keep the cells in this condition for 3 passages and then reduce the FBS to 2 %. At this point, cells are ready.
- Cells should be split at a ratio of 1:5 every 2 days or 1:10 every 3 days. Avoid allowing them to reach over confluency.

### Influenza virus propagation in MDCK cells

#### Timing: 48–72 h

- Remove the media from a MDCK culture flask in which the monolayer has reached approximately 80-90 % confluency.
- Wash the monolayer with PBS.
- Add 5 mL of OptiMEM 1 % PS/0.5 % BSA containing virus at a dilution that makes a MOI of approximately 0.1.
- Incubate for 1 h at 37°C/5 % CO2, rocking the flask every 10-15 min.
- Discard the infectious media and add OptiMEM 1 % PS/0.5 % BSA/1-2.5 μg/mL trypsin.
- Incubate the flasks at 37°C/5 % CO2 and follow the course of the infection by inspecting the cells under the microscope. Proceed to harvest when most of the cells show cytopathic effect (48-72 h postinfection).
- Clarify the supernatant by centrifugation at approximately 230 g for 10 min.
- Collect the supernatant, make aliquots and store them at −80°C for use in the antiviral assay.

### Virus titration by CPE assay

Determining the dilution of the virus stock that produces CPE in 50 percent of the cells (tissue culture infectious dose 50: TCID50) is required to estimate the amount of virus in the stock, and it is performed every time a new stock is produced. TCID50/mL values between 10,000 and 100,000 are expected for most influenza viruses.

#### Timing: 72 h

##### 1. MDCK harvest

- Remove the culture media and wash the cells with PBS.
- Add 5 mL of TrypLE Express to the flask and incubate for 2 min at RT.
- Remove the TrypLE Express and incubate the flasks at 37 °C/5 % CO2 for 8 min.
- Add 10mL of OptiMEM/10 % FBS, collect and transfer the cells to a 50 mL conical tube.
- Add additional 5-10 mL of OptiMEM/10 % FBS to recover the remaining cells. Use a scraper if needed.
- Centrifuge the cells at approximately 230 g for 4 min.
- Discard the media, wash the cells with OptiMEM/1 % PS and centrifuge.
- Resuspend cells in OptiMEM 1 % PS and count.
- Prepare the cell suspension for the assay to add 5000 cells/well in OptiMEM 1 % PS, 0.5 % BSA/1-2.5 μg/mL trypsin.

##### 2. Titration

**Fig. 1.**
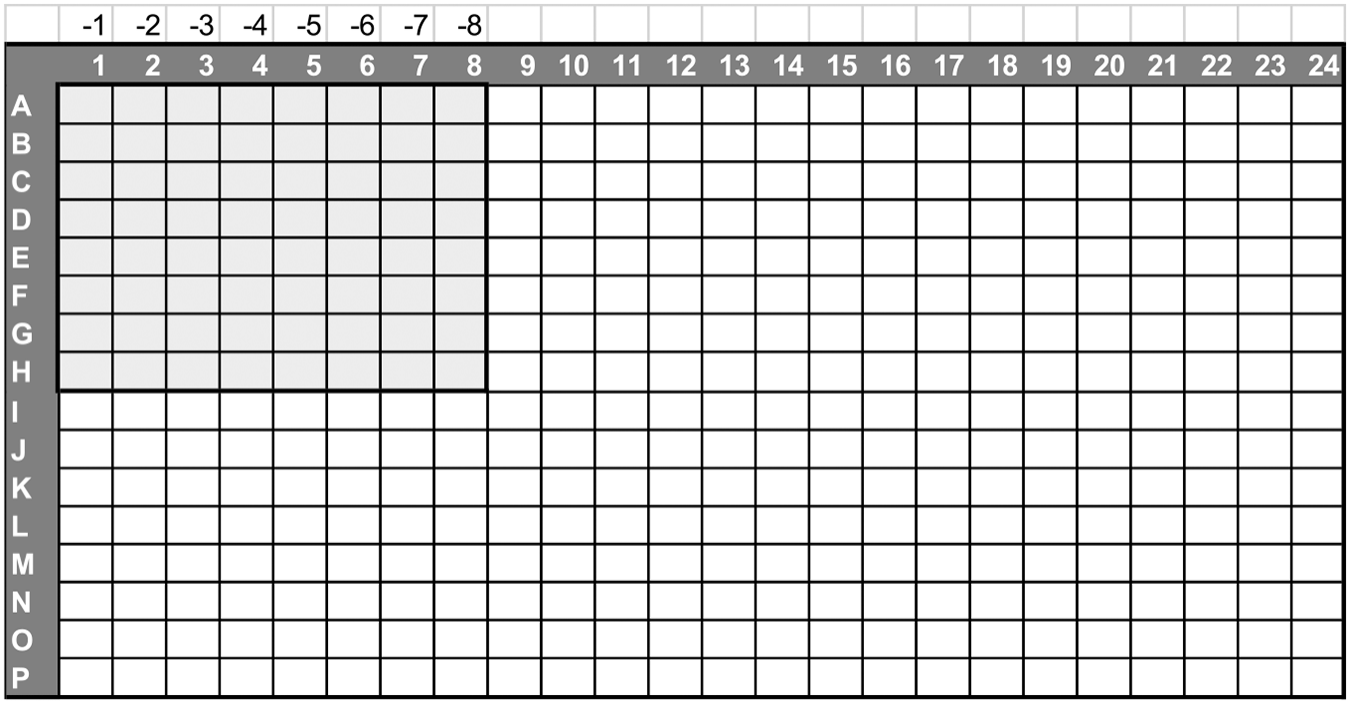
Virus dilution plate layout. Labels over columns 1 to 8 display log of dilutions of virus.

**Fig. 2.**
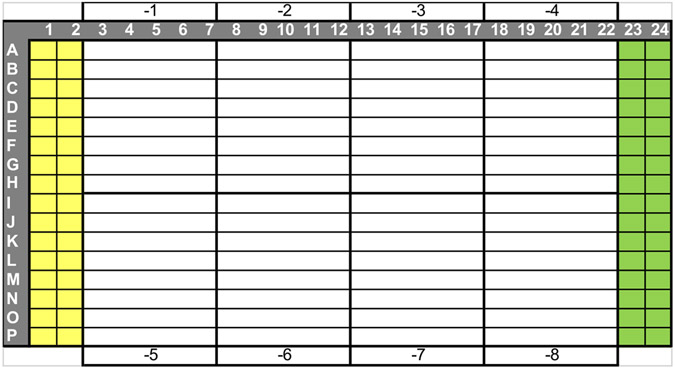
Titration assay plate layout. Virus dilutions are to be added to each section of the plate using an automatic multichannel pipette going from the bottom right to the plate (8 log dilution) to the top left (1 log dilution). Columns 1 & 2 and 23 & 24 are the assay cell and virus control, respectively.

- Prepare a plate for performing log dilutions of the virus stock by adding 45 μL of OptiMEM 1 % PS to eight columns and eight rows (Fig. 1). Use an Axygen polypropylene plate or any other non-binding plate.
- Add 5 μL of the influenza stock to the 8 wells in column 1.
- Using an automatic multichannel pipette, start making log dilutions by transferring 5 μL from one column to the other, mixing and changing tips between each transfer.
- Following the layout in Fig. 2, add 5 μL of the virus dilutions to the titration plate with an automatic multichannel pipette, starting with the −8 dilution (no tips change needed between dilutions). Each section of the titration plate corresponds to the log dilution in each column of the dilution plate.
- Add 5 μL of OptiMEM 1 % PS to cell control wells in columns 1 and 2 (high signal control, cells with no virus).
- Make a 1:50 dilution of the virus stock in OptiMEM 1 % PS and add 5 μL to infected cell control wells in columns 23 and 24 (low signal control, cells plus virus).
- Add 25 μL of MDCK cells at 200,000 cells/mL in OptiMEM 1 % PS/6 % BSA/1.2-3 μg/mL trypsin to all wells. Final BSA percentage and trypsin concentration are 0.5 and 1-2.5 μg/mL, respectively.
- Incubate for 72 h at 37 °C/5 % CO2/high humidity.
- Equilibrate plates and CellTiter-Glo to room temperature (RT).
- Dispense same volume of CellTiter-Glo as the volume in the well.
- Incubate for 10 min at RT for signal stabilization.
- Read the luminescence using a microplate reader.

### Antiviral assay

**Fig. 3.**
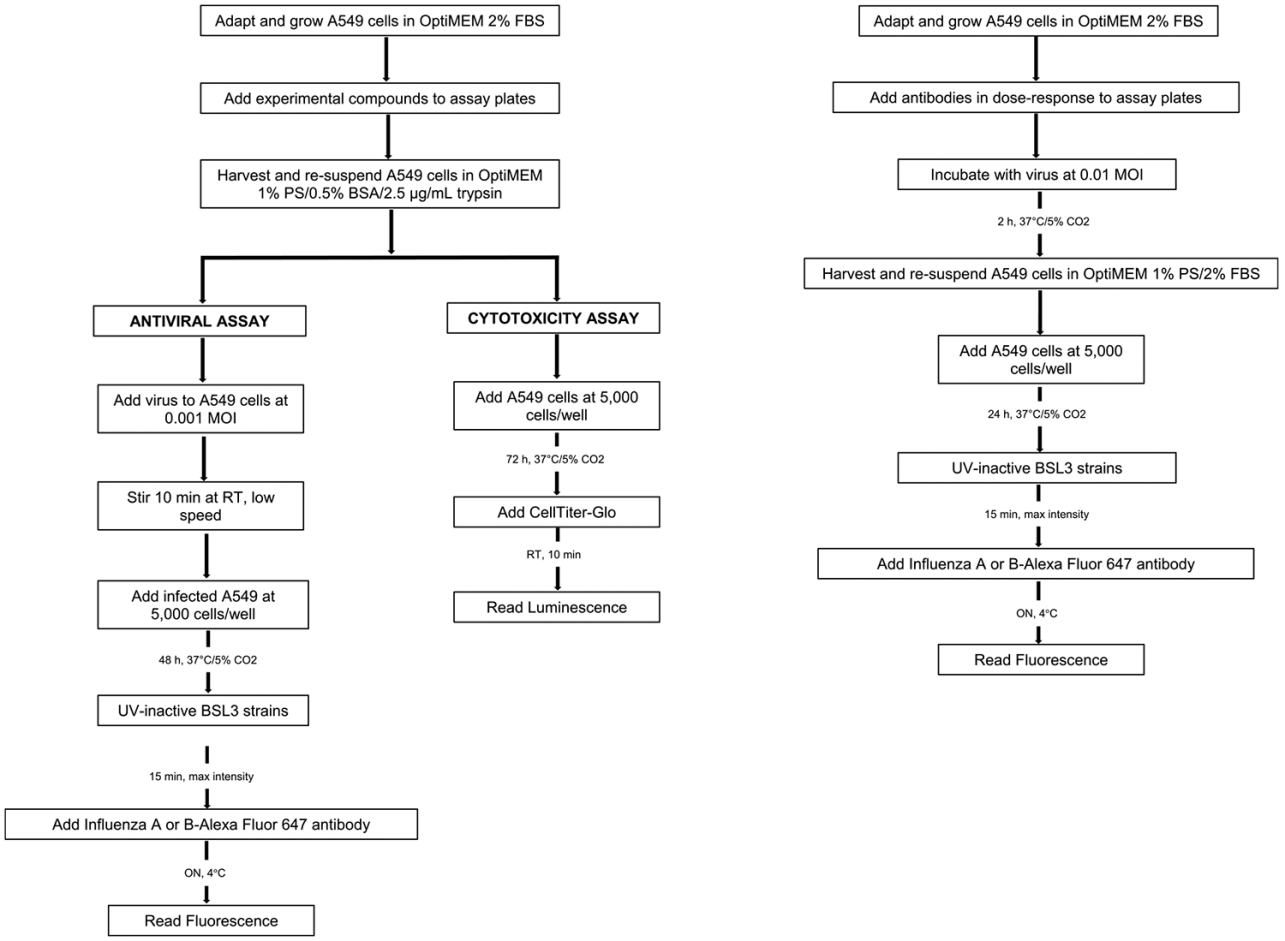
Flow diagram of influenza immunofluorescence assay for detection of inhibitors of any stage of the virus life cycle (left panel) and for detection of the blocking activity of anti-influenza antibodies (right panel).

Two approaches of the antiviral assay have been designed. One involves multiple virus infection and replication cycles allowing the identification of anti-influenza compounds that inhibit any stage of the virus life cycle. The other one has been developed to assess the ability of anti-influenza antibodies to block the interaction of virus-cell receptor. Both approaches have been sumarized in the flow diagram shown in Fig. 3.

### Antiviral assay for detection of inhibitors of any stage of the virus life cycle

#### Timing: 72 h

##### 1. Drugging of antiviral assay plates

- Solubilize experimental compounds dry powder in DMSO to make 10 mM stock concentrations.
- Add 80 μL of each 10 mM sample stock to a single well of column 3 or column 13 of the source plate.
- Add 40 μL of DMSO to the remaining wells in the source plate.
- Create a 10-point concentration range for each compound by transferring 40 μL of the 10 mM per well samples in column 3/13 across the corresponding rows through column 12/22. The concentration range within the source plate for each sample is as follows: 10 mM, 5 mM, 2.5 mM, 1.25 mM, 0.625 mM, 0.3125 mM, 0.1563 mM, 0.0781 mM, 0.0391 mM, 0.0195 mM.
- Centrifuge at 60 xg for 1 minute to remove any bubbles from the wells.
- Using an acoustic liquid transfer system, transfer 120 nL from the wells in the source plate to the same wells in the assay plate. Final dilution ratio of the 10 mM compound stocks is 1:250 (120 nL sample into 30 μL total assay volume) for a final high-test concentration of 40 μM. The test concentration range for each sample is as follows: 40 μM, 20 μM, 10 μM, 5 μM, 2.5 μM, 1.25 μM, 0.625 μM, 0.3125 μM, 0.1563 μM, and 0.0781 μM
- Follow the same steps to create a reference compound source plate and to transfer them to the assay plate. The 10-point concentration range for each reference compound is as follows:
  - VX-787: 40 nM, 20 nM, 10 nM, 5 nM, 2.5 nM, 1.25 nM, 0.625 nM, 0.3125 nM, 0.1563 nM, 0.0781 nM
  - Ribavirin: 100 μM, 50 μM, 25 μM, 12.5 μM, 6.25 μM, 3.125 μM, 1.5625 μM, 0.7813 μM, 0.3906 μM, 0.1953 μM
  - Oseltamivir: 500 nM, 250 nM, 125 nM, 62.5 nM, 31.25 nM, 15.625 nM, 7.8125 nM, 3.9063 nM, 1.9531 nM, 0.9766 nM
- Transfer 120 nL of DMSO to cell control wells in columns 1, 2 and to virus control wells in columns 23, 24 rows from A to L.
- Transfer 120 nL of positive control VX-787 to columns 23 and 24, rows M-P for a test concentration of 20 nM.

##### 2. Drugging of cytotoxicity assay plates

- Follow the procedure previously described to drug the cytotoxicity assay plates from the same experimental and reference compounds source plates.
- Transfer 120 nL of DMSO to cell control wells in columns 1, 2, 23 and 24. Columns 1 and 2 are the high signal control wells (cells only, no compound) and columns 23 and 24 are the low signal control wells to which hyamine is later added to a final concentration of 100 μM.

##### 3. A549 harvest

- Remove the culture media and wash the cells with PBS.
- Add 5 mL of TrypLE Express to the flask and incubate for 2 min at RT.
- Remove the TrypLE Express and incubate the flasks at 37 °C/5 % CO2 for 4 min.
- Add 10mL of OptiMEM/10 % FBS, collect and transfer the cells to a 50 mL conical tube.
- Add additional 5-10 mL of OptiMEM/10 % FBS to recover the remaining cells. Use a scraper if needed.
- Centrifuge the cells at 230 xg for 4 min.
- Discard the media, wash the cells with OptiMEM/1 % PS and centrifuge.
- Resuspend cells in OptiMEM 1 % PS and count.
- Prepare the cell suspension for the assay to add 5000 cells/well in OptiMEM 1 % PS, 0.5 % BSA/1-2.5 μg/mL trypsin

##### 4. Antiviral assay

**Fig. 4.**
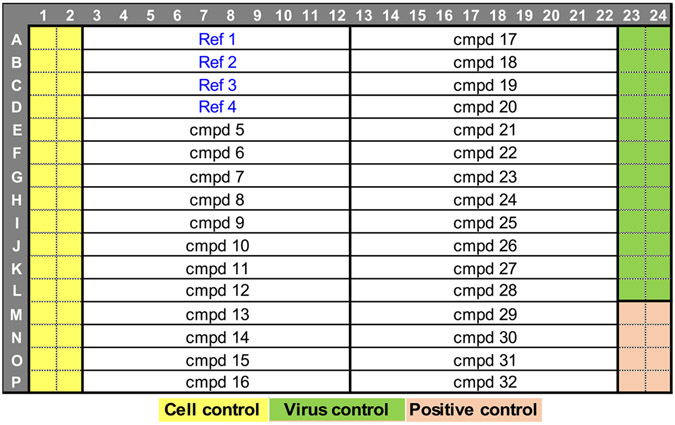
Antiviral assay plate layout. Up to 32 compounds can be tested per assay plate. Replicate occurs in a separate plate.

- Split the cell suspension into 2 volumes enough for cell control wells (columns 1 and 2) and infected wells (columns from 3 to 24). Plate layout is shown in Fig. 4.
- Add influenza virus at a MOI of 0.001 to the cell suspension for addition to infected wells and stir at a low speed for 10 min. Virus volume should be small enough in order to not significantly change the cell density.
- To the pre-drugged plates, dispense cells only to columns 1 and 2 and infected cells to columns 3 to 24. Final volume per well is 30 μL.
- Centrifuge the plates 5 min at approximately 230 xg.
- Incubate for 48 h at 37 °C/5 % CO2/high humidity.
- If the influenza strain requires BSL3 containment, inactivate the virus using ultraviolet radiation by exposing the plates without the lid to the maximum intensity (~8000 μW/cm2) of a Spectrolinker XL-1500 and removed the plates from the BSL3 facility.
- Decant the supernatant by flipping the plate over a tray with paper towels imbedded in disinfectant, and gently tap it over a cushion of dry paper towels.
- Using a bulk liquid dispenser, add 20 μL of the influenza M2 antibody-Alexa Fluor 647 at 1:1000 dilution in OptiMEM/1 % PS/2 % BSA or influenza B antibody-Alexa Fluor 647 at a 1:500 dilution in OptiMEM/1 % PS/2 % BSA.
- Centrifuge 1 min at 230 xg.
- Incubate at 4°C for about 18-20 h.
- Seal the plates and read the fluorescence signal using a laser scanning cytometer.

##### 5. Cytotoxicity assay_Counterscreen

- Add hyamine to columns 23 and 24 for a final concentration of 100 μM.
- Dispense 5000 A549 cells suspended in OptiMEM 1 % PS/0.5 % BSA/1-2.5 μg/mL trypsin to all the wells of the pre-drugged assay plates for a final volume per well of 30 μL.
- Incubate for 72 h at 37 °C/5 %CO2/high humidity.
- Equilibrate plates and CellTiter-Glo to RT.
- Dispense 30 μL of CellTiter-Glo to entire plate and incubate for 10 min at RT for signal stabilization.
- Read the luminescence using a microplate reader.

### Antiviral assay for detection of the blocking activity of anti-influenza antibodies

#### Timing: 48 h

This approach of the assay allows the detection of inhibitors of the virus entry and genome replication only. It has been specifically designed for the assessment of the ability of anti-influenza antibodies to block the interaction of virus-cell receptor. As only one cycle of replication is needed, the addition of trypsin has been eliminated, which additionally allows the testing of the inhibitory potency of antibodies in serum. A MOI that is at least 10 times higher than the one used for the detection of inhibitors of any stage of the virus life cycle is required.

##### 1. Drugging of plates

- Dilute purified experimental and control antibodies in PBS to 6 times the highest test concentration to be used in the assay.
- Add 75 μL of the 6x diluted stock to a single well of column 3 or column 13 of the source plate. Use an Axygen polypropylene plate or any other non-binding plate.
- Add 60 μL of PBS to the remaining wells of the source plate.
- Create a 10-point concentration range for each antibody by transferring 15 μL of the 75 μL 6x samples in column 3/13 across the corresponding rows through column 12/22. If the concentration range within the source plate for control/experimental antibody starts at 75 μg/mL (high test concentration), the full dose-response range is as follows: 75 μg/mL, 15 μg/mL, 3 μg/mL, 0.6 μg/mL, 0.12 μg/mL, 0.024 μg/mL, 4.8 ng/mL, 0.96 ng/mL, 0.192 ng/mL, 0.0384 ng/mL
- Using tip-based automated liquid handling with a 384-well multichannel head, transfer 5 μL of assay media to all the wells of the assay plates.
- Transfer 5 μL of the serially diluted control antibodies from the source plate to the assay plates. The assay plates now contain 10 μL total volume. With the addition of 5 μL of virus along with 15 μL of cells, the total assay volume per well is 30 μL resulting in a final dilution of the control antibodies of 1:6.
- Transfer 5 μL of assay media to cell control wells in columns 1, 2 and to virus control wells in columns 23, 24 rows from A to L.
- Transfer 5 μL of positive control antibody MD3606 to columns 23 and 24, rows M-P for a test concentration of 40 nM.

Note: Step e was conceived to enable the comparison of the inhibitory potency of the antibodies when purified and solubilized in PBS and when in serum sampled from immunized animals. If this comparison is desired, 5 μL of non-immunized animal serum would be added in this step for purified antibodies. When testing serum samples, 5 μL of media are added.

#### A549 harvest

- Remove the culture media and wash the cells with PBS.
- Add 5 mL of TrypLE Express to the flask and incubate for 2 min at RT.
- Remove the TrypLE Express and incubate the flasks at 37 °C/5 % CO2 for 4 min.
- Add 10mL of OptiMEM/10 % FBS, collect and transfer the cells to a 50 mL conical tube.
- Add additional 5-10 mL of OptiMEM/10 % FBS to recover the remaining cells. Use a scraper if needed.
- Centrifuge the cells at 230 xg for 4 min. Discard the media and resuspend cells in OptiMEM 1 % PS/2 % FBS and count.
- Prepare the cell suspension to add 5000 cells/well in OptiMEM 1 % PS/2 % FBS.

##### 2. Antiviral assay

- Add 5 μL of media to columns 1 and 2 (cell control wells, no antibody).
- Add 5 μL of virus 6 times the dilution previously determined to be used in the assay to columns 3 to 24. Plate layout is same as the one previously shown.
- Incubate for 2 h at 37 °C/5 % CO2/high humidity.
- Harvest, wash and re-suspend the cells in OptiMEM 1 % PS/2 % FBS to add 5000 cells/well/15 μL. Final volume per well is 30 μL.
- Add cells to all the wells.
- Centrifuge 5 min at 230 xg.
- Incubate for 24 h at 37°C/5 % CO2/high humidity.
- If the influenza strain requires BSL3 containment, inactivate the virus using ultraviolet radiation by exposing the plates without the lid to the maximum intensity (~8000 μW/cm2) of a Spectrolinker XL-1500 and removed the plates from the BSL3 facility.
- Decant the supernatant by flipping the plate over a tray with paper towels imbedded in disinfectant and gently tap it over a cushion of dry paper towels.
- Using bulk liquid dispenser, add 20 μL of the influenza A M2 Ab-Alexa Fluor 647 at 1:1000 dilution or influenza B antibody-Alexa Fluor 647 at a 1:500 dilution in OptiMEM/1 % PS/2 % BSA.
- Centrifuge 1 min at 230 xg.
- Incubate at 4°C for about 18-20 h.
- Seal the plates and read the fluorescence signal using a laser scanning cytometer.

### Data processing

#### Percentage of inhibition calculation

For calculation of the percentage of inhibition, the raw signal data for each well is normalized to percentage of inhibition by the following formula:

$$\%\;inhibition\;=100^{*}[(test\;compound-avg\;virus\;signal)\;∕\;(avg\;cell\;signal-avg\;virus\;signal)]$$

IC50 values are calculated by a four-parameter logistic fit of % inhibition versus log[compound] with the minimum parameter fixed at 0 and maximum parameter fixed at 100.

#### Percentage of viability calculation

For calculation of the percentage of viability, the raw signal data for each well was normalized to percentage of viability by the following formula:

$$\%\;viability\;=\;100^{*}[(test\;compound-avg\;positive\;control\;signal)\;∕\;(avg\;cell\;signal-avg\;positive\;contro\;signal)]$$

CC50 values are calculated by a four-parameter logistic fit of % viability versus log[compound] with the minimum parameter fixed at 0 and maximum parameter fixed at 100.

#### TCID50 calculation

The percentage of viability relative to the cell control is calculated for each well. Each tested well is assigned a score of 0 if the viability is greater than 50 %, or a score of 1 if it is less than 50 %. Scores of all wells for each virus dilution are added together for a “Sum” of the virus positive wells. Any fit containing at least one “Sum” that is greater than half of the number of replicates for that virus dilution is considered “Active”. The Sum (y) of each log dilution (x) is plotted using the four-parameter logistic equation below, where A is the minimum y value, B is the maximum y value, C is the IC50 value and D is the slope factor.


$$y=(A+(B-A)\;∕\;(1+{\left(x∕C\right)}^{\wedge }D))$$

#### Critical assay considerations/steps

**Fig. 5.**
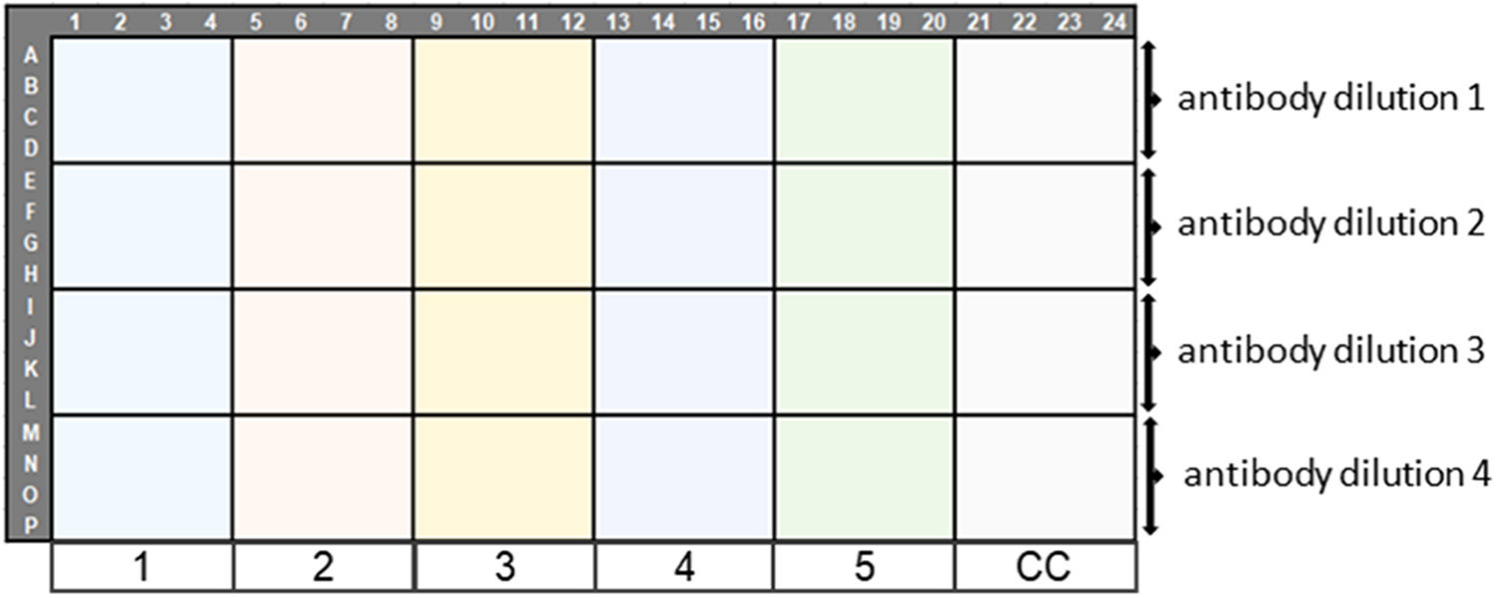
Plate layout for combining virus titration in the immunofluorescence assay and ability of the antibody to detect the strain of interest. 1 to 5 represents 5 virus dilutions and CC is for cell control (no virus).

**Fig. 6.**
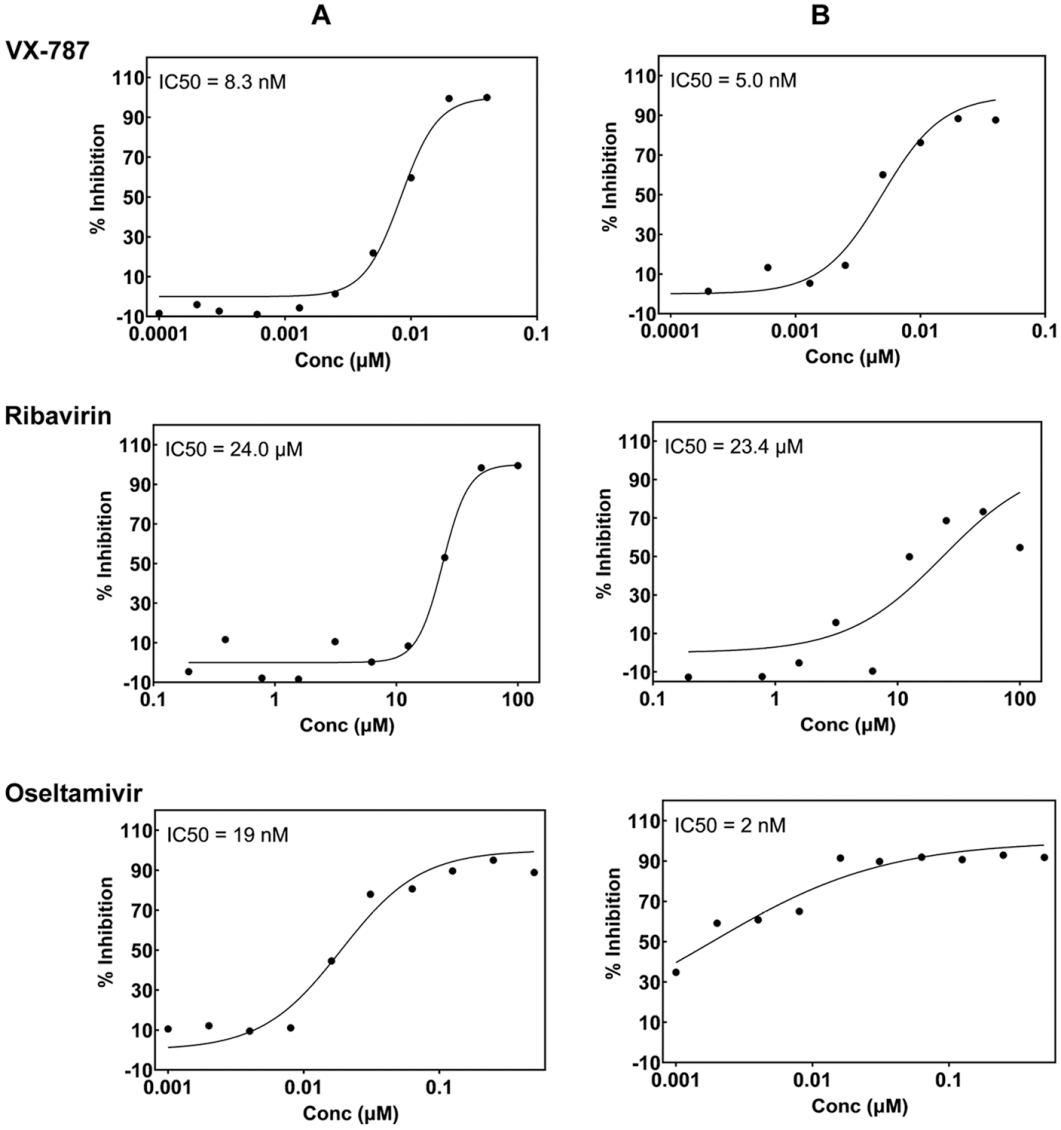
Dose-response of reference compounds obtained from the data produced by (Panel A) fluorescence readout and (Panel B) CPE readout. Assay variant is the one for detection of inhibitors of any stage of the virus life cycle and virus strain is A/Udorn/72, H3N2.

1. To determine the concentration of trypsin that activates the virus HA without significantly affecting cell attachment, a titration must be run every time the trypsin is prepared whether from the same powder lot or from a different one. Changing the trypsin provider could have a negative impact on the assay. It may be necessary to evaluate trypsin from different sources.
2. BSA solution should be tested in the assay every time it is prepared from a different product lot. Changing the BSA provider could have a negative impact on the assay. If obtaining the BSA from a different provider is necessary, a titration to determine the % that can be added to the assay media without affecting the virus HA cleavage by the trypsin must be run. It may be necessary to evaluate BSA from different sources.
3. After obtaining the TCID50 through CPE, the virus dilution to be used in the immunofluorescence assay must be precised by running another titration through the immunofluorescence assay. A closer range of virus dilutions around a value that is 100 times the determined TCID50 should be tested. For example, dilutions of 1:5000, 1:7500, 1:10,000, 1:25,000 and 1:50,000 for a strain with a TCID50/mL of 10^6^. Once the dilution for the immunofluorescence assay has been determined, a final test with reference compounds must be run. The IC50 values of the reference compounds obtained should be comparable with those reported in the literature.
4. The ability of the antibody selected for detection of the strain of interest has to be tested. An experiment combining the titration in the immunofluorescence assay and the determination of the antibody’s ability to detect, as well as its optimal dilution, is recommended. An example of the plate layout is shown in Fig. 5.
5. The inactivation through ultraviolet radiation of the BSL3 influenza strains has to be validated and approved by the institutional responsible official (RO).
6. When using a bulk liquid dispenser for the addition of the detection antibody, the instrument should be set for adding to the side of the well at a medium speed in order to avoid dislodging the cells.
7. Supernatant removal and detection antibody addition steps can be automated using a tip-based automated liquid handling with a 384-well multi-channel head with similar results. Instrument should be set for removing the supernatant and adding the antibody at a low speed and to the corner of the well without touching the bottom. Tips are rinsed with PBS between steps and decontaminated with an approved disinfectant at the end of the run.
8. The fluorescence readout can be combined with a CPE readout using CellTiter-Glo if the incubation of cells with the detection antibody is carried out at 37°C/5 % CO2 instead of at 4°C (step 4. r of assay for detection of inhibitors of any stage of the virus life cycle and step 3.p of assay for detection of the blocking activity of anti-influenza antibodies), by adding 20 μL of CellTiter-Glo after the fluorescence read is completed. The overnight incubation of the cells with the detection antibody at 37°C/5 % CO2 does not modify the fluorescence readout (Fig. 6).
9. The use of collagen coated plates for both antiviral and cytotoxicity assays is required. A549 cells have shown sensitivity to the presence of trypsin in the assay media, and the coat of collagen contributes to the attachment and hence maintenance of the cell morphology.
10. The cytotoxicity assay should be setup the same day as the antiviral assay using the same cell suspension.
11. Percentage of DMSO should be same in all wells and under 0.4, including control wells.
12. Biosafety measures for *in vitro* work with influenza virus are to be followed as established by the institutional Environmental Health and Safety department.

#### Troubleshooting

Problems that cause the antiviral assay to fail are summarized in Table 3, alongside the potential cause, and corrective action for each.

## 3. Anticipated results

Two approaches of a protocol for an imaging assay for detection of inhibitors of influenza virus using a human cell line that is HTS compatible are provided here. One of the protocols allows the identification of anti-influenza compounds that inhibits any stage of the virus life cycle, while the other one is designed for evaluation of the potency of anti-influenza antibodies to block the interaction virus-cell receptor.

**Fig. 7.**
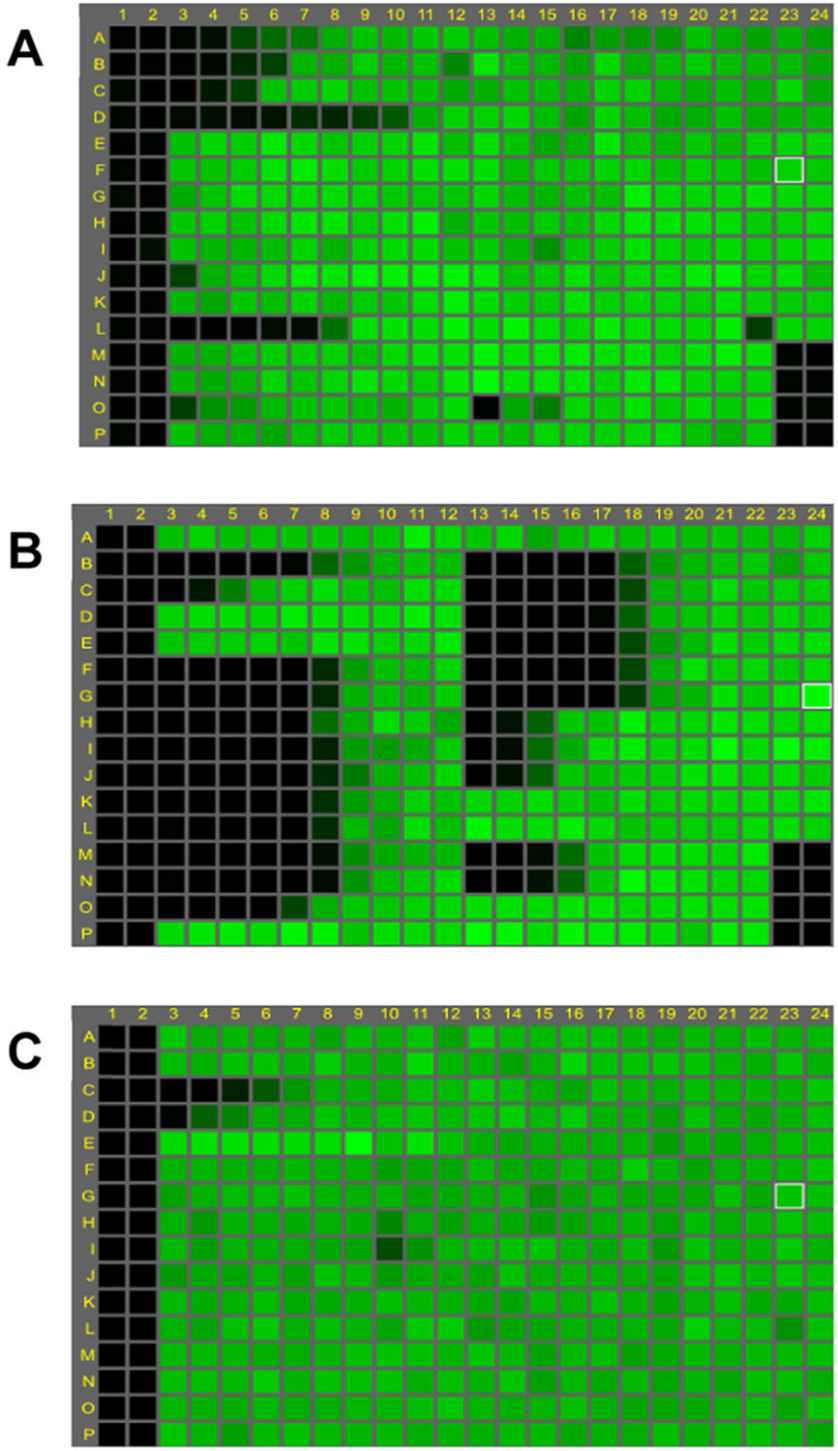
Fluorescence maps of results from assay for detection of (A) inhibitors of any stage of the virus life cycle and (B) blocking activity of anti-influenza antibodies. Columns 1 & 2 contain cells only for low signal control and columns 23 & 24, rows A to L contain cells plus virus (no compound) for high signal control. Columns 23 & 24 from M to P are the assay positive control drug (A) VX787 at 20 nM and (B) antibody control MD3606 at 40 nM. High concentration of test compounds/antibodies are in column 3 and 13. Dose response of reference compounds VX-787, ribavirin and oseltamivir are in columns 3 to 12 rows B, C and D, respectively. C is the map of the small molecule reference compounds run alongside test antibodies; VX-787, ribavirin and oseltamivir are located in rows C, D and E; oseltamivir inhibitory activity is not detected as the assay for antibody testing only allows for one replication cycle, therefore unable to detect virus egress inhibitors. Virus strains: (A) A/Udorn/72, H3N2; (B & C) A/mallard/Netherlands/12/00, H7N7.

**Fig. 8.**
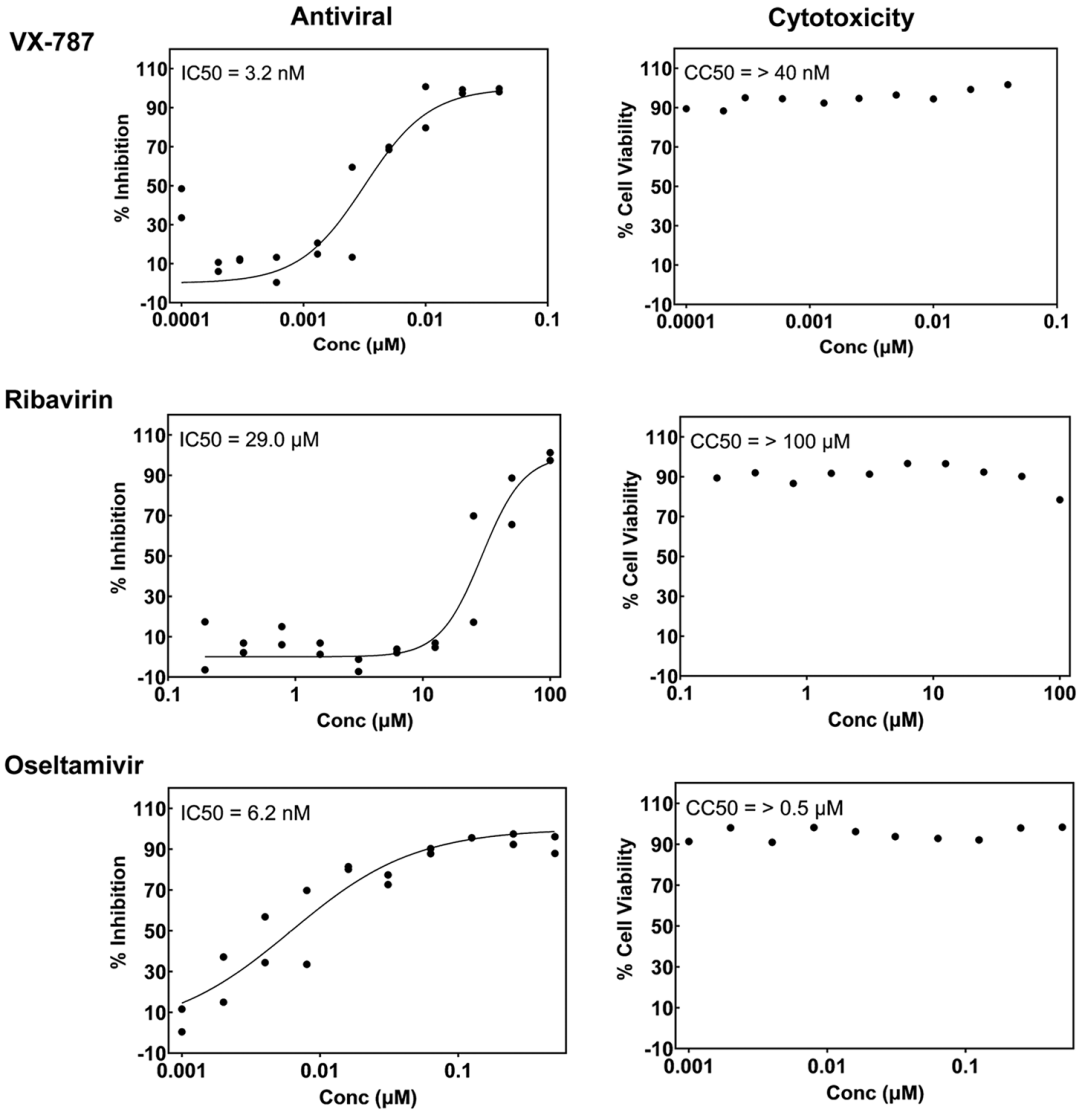
Dose-response of reference compounds tested in duplicate in the assay for detection of inhibitors of any stage of the virus life cycle against A/Udorn/72 (H3N2) and in singlet in the cytotoxicity assay. Concentration that produces inhibition of the virus in 50 percent of the cells (IC50) and that is cytotoxic for the 50 percent of the cells (CC50) are shown in the top left corner of the graphs. High dose is 40 nM for VX-787, 100 μM for ribavirin and 0.5 μM for oseltamivir.

To evaluate the quality of the assay, influenza inhibitors VX-787, ribavirin and oseltamivir are included as references in each run of the assay variant for detection of inhibitors of any stage of the virus life cycle (Fig. 7). A single inhibitory dose of VX-787 is also included in each assay plate as positive control. For the assay to detect the blocking activity of influenza antibodies, SD36, SD38, SD83, SD84 and MD3606 are used as reference [12]. Positive control antibody is MD3606, which is active against influenza A and B. IC50 values of reference compounds/antibodies, 100 % inhibition of the positive control drug and Z’ values higher than 0.5 are used as the criteria to accept the data and continue to the processing step. IC50 values obtained for VX-787, oseltamivir and ribavirin in both variants of the assay and IC50 values obtained for reference antibodies are shown in Tables 4 and 5, respectively. Dose-response curves of reference compounds of the multicycle variant of the assay are shown in Fig. 8.

**Fig. 9.**
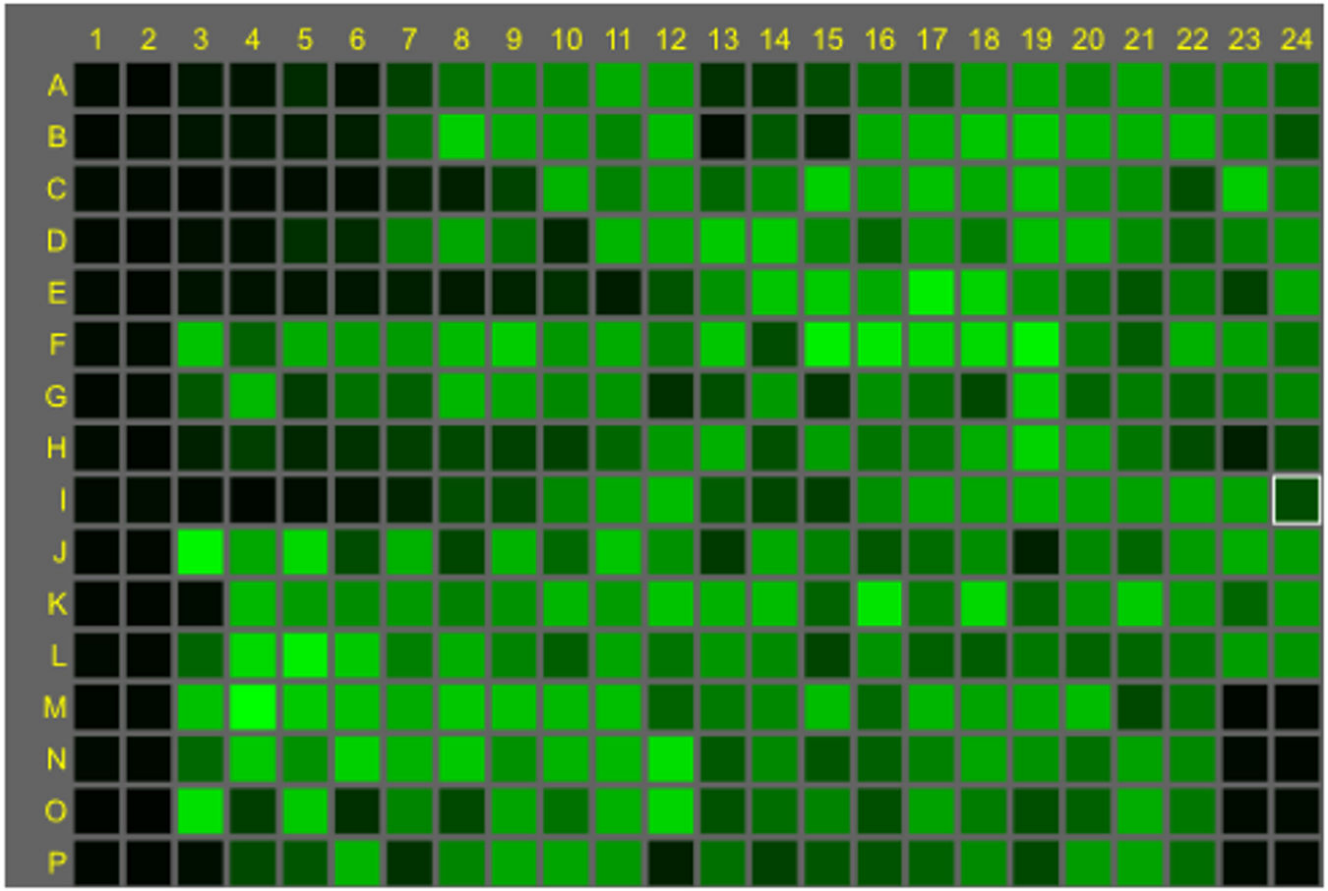
Fluorescence map of assay for detection of inhibitors of any stage of the virus life cycle showing variable infection due to the addition of insufficient trypsin. The assay media contained 1.5 μg/mL of trypsin. A troubleshooting trypsin titration experiment run after this result showed the trypsin stock had lost potency and its concentration for the assay was increased to 2.5 μg/mL. A new stock was prepared that is being used at 1 μg/mL. Virus strain: A/Udorn/72, H3N2.

The addition of trypsin in order to activate the virus HA of most influenza strains constitutes the most challenging requirement of cell-based antiviral influenza assays that involve multiple rounds of virus replication. The concentration of trypsin that activates the virus without significantly impacting cell attachment has to be determined through a very careful titration. No infection or variable infection will be observed if insufficient trypsin is added to the assay media (Fig. 9). The trypsin will not cleave the virus HA if FBS is present in the assay media, making it essential to adapt the cell growth to a low percentage of FBS in the culture media. The addition of trypsin is not required for assessment of the blocking activity of anti-influenza antibodies as a multi cycle assay is not necessary for the detection of this activity, allowing serum samples to be tested.

This methodology is available as a tool to determine the breadth of antiviral activity of experimental compounds across multiple influenza strains, allowing the identification of broader spectrum inhibitors in an early stage of the drug discovery. Also, the possibility of combining the fluorescence and CPE readouts, provides an avenue for identifying small molecules whose actions probe the relationship between virus replication and cellular response.

## Figures and Tables

**Table 1:** Advantages and disadvantages of cell-based influenza assays used in HTS.

Assay	Challenges/Disadvantages	Advantages
Cytopathic effect (CPE) assays [3,4,5]	Indirect measure of virus growth and, therefore, higher likelihood of false positive or negative results Requires trypsin to achieve multicycle virus growth (most seasonal strains) Constrained by cytotoxicity Prone to variability Mostly restricted to the use of MDCK cells	Allow infection at a low multiplicity of infection (MOI) and therefore able to detect inhibitors of any virus cycle stage Ability to select small molecules that are membrane permeable, stable and functional in a cellular environment.
Virus inducible reporter assays [6,7,8]	Need high MOI to achieve reporter activation preventing the detection of virus egress inhibitors Require generation of a stable cell line or choice of cell with a high transfection efficiency.	Direct measure of virus infection Useful to quantify virus in supernatants
Reporter-encoding influenza virus assays [9,10,11]	Require an efficient reverse genetics system and knowledge of packaging signals Competence for packing of the reporter segment with the viral segment that contains the same packing sequences Require a compliment cell line if multiple cycles of replication are desired.	Direct measure of virus infection Any cell line susceptible to influenza infection can be used

**Table 2:** Timing of specific stages within major steps.

Stage	Timing
Cell harvest	30 min
Infection for growing virus	1 ¼ h
Compound source plate generation	1-1 ½ h
Plate drugging	15-30 min per 10 plates
Assay setup	30 min for first 10 plates, then 15 min per 10 plates
Supernatant removal and antibody addition using a bulk liquid dispenser	10 min per 10 plates
Supernatant removal and antibody addition, both using a tip-based automated liquid handling	30 min per 10 plates

**Table 3:** Guide to assay troubleshooting.

Problem	Potential cause	Protocol step	Corrective action
Plating pattern (striping pattern, checkerboard pattern)	Dispensing cassette tip obstruction during dispensing	Cells and cells plus virus dispensing	Obtain a new dispensing cassette
	Dispenser failure	Cells and cells plus virus dispensing	QC dispensing instrument
Edge effect	Wrong incubator humidity setting	Incubation	Ensure the incubator humidity is 95%. For added humidity, incubate the plates inside a pan with dampened paper towels
Low variable signal	Loss of trypsin activity during storage	Antiviral assay setup	Re-titration of the trypsin working stock / preparation of a new stock
	Decrease in virus titer during storage	Antiviral assay setup	Re-titration of the virus stock
	Bad batch of collagen coated plates	Antiviral assay setup	Rerun in parallel with a plate from a different batch and obtain a new batch of plates

**Table 4:** IC50 (μM) values of reference compounds tested against different influenza strains using the assay for detection of (A) inhibitors of any stage of the virus life cycle and (B) blocking activity of anti-influenza antibodies. For A/WSN/33, VX787 and Ribavirin show similar IC50 values under both assay conditions, while Oseltamivir’s IC50 is 10 times higher when tested in the assay to assess the potency of antibodies for blocking the virus entry. Oseltamivir potency could not be determined for the rest of the strains in the concentration range used in the assay for detection of the blocking activity of antibodies. Results in Table A are the average of at least 7 determinations.

A. Influenza strain	VX-787	Ribavirin	Oseltamivir
A/WSN/33, H1N1	0.008	33.72	0.04
A/Udorn/72, H3N2	0.01	33.18	0.07
B. Influenza strain	VX-787	Ribavirin	Oseltamivir
A/WSN/33, H1N1	0.0037	33.2	0.135
A/Brisbane/10/07, H3N2	0.013	64.67	>0.5
A/mallard/Netherlands/12/00, H7N7	0.0049	43.55	>0.5
B/Bris/60/2008, Victoria lineage	>0.04	9.69	>0.5
B/Florida/4/2006, Yamagata lineage	>0.04	2.69	>0.5

**Table 5:** IC50 values of reference control anti-influenza antibodies tested against different influenza virus strains using the assay for detection of the blocking activity of anti-influenza antibodies. Results are in agreement with Laursen et al., 2018.

Influenza strain	IC50 nM SD36	SD38	SD83	SD84	MD3606
A/WSN/33, H1N1	7.5	0.2	>0.0005	>0.0005	0.09
A/Brisbane/10/07, H3N2	1.3	4.4	>0.0005	>0.0005	2
A/mallard/Netherlands/12/00, H7N7	0.11	25	>0.0005	>0.0005	ND
B/Bris/60/2008, Victoria lineage	>0.0005	>0.0005	94	0.63	1.1
B/Florida/4/2006, Yamagata lineage	>0.0005	>0.0005	>0.0005	0.97	0.7

ND: Not determined.
